# Divergent Prognostic Impact of Histopathological Features and Combined Hematological–Biochemical Indices in Rectal Versus Colorectal Cancer: A Single-Center Study

**DOI:** 10.14740/wjon2787

**Published:** 2026-06-25

**Authors:** Vladica Cuk, Jovan Juloski, Marijana Gacinovic, Mirela Cuk, Radosav Radulovic, Jelena Grahovac

**Affiliations:** a “Nikola Spasic” Surgical Clinic, Zvezdara University Medical Center, Belgrade, Serbia; b Faculty of Medicine, University of Belgrade, Belgrade, Serbia; c Clinic for Internal Medicine, Zvezdara University Medical Center, Belgrade, Serbia; d Department of Experimental Oncology, Institute for Oncology and Radiology of Serbia, Belgrade, Serbia

**Keywords:** Colorectal cancer, Rectal cancer, Clinicopathologic features, Hematological/biochemical indices

## Abstract

**Background:**

Colorectal cancer is frequently analyzed as a single disease entity despite recognized biological and clinical differences between colon and rectal tumors. This study investigated whether systemic inflammatory and nutritional indices demonstrate similar prognostic value in a pooled colorectal cancer cohort and in rectal adenocarcinoma analyzed separately.

**Methods:**

A predefined subgroup analysis of a previously established single-center cohort of surgically treated patients with long-term follow-up was performed. Prognostic associations of clinical, hematological, biochemical, and histopathological variables were evaluated in the overall colorectal cancer cohort and subsequently in patients with rectal adenocarcinoma using Kaplan–Meier and Cox regression analyses.

**Results:**

In the colorectal cancer cohort, severe postoperative complications (Clavien–Dindo III–V), higher lymph node ratio, tumor deposits, and modified Glasgow Prognostic Score 2 were independently associated with worse overall survival, while TNM stage, reduced peritumoral lymphocytic response, and tumor deposits predicted disease-free survival. In contrast, rectal adenocarcinoma demonstrated a distinct prognostic pattern: perineural invasion was associated with poorer overall survival in the exploratory rectal cancer subgroup analysis (hazard ratio (HR) = 25.125; P = 0.003), whereas platelet-to-lymphocyte ratio retained statistical significance with a modest effect size. Other systemic inflammatory and pathological parameters showed limited impact.

**Conclusions:**

Colon and rectal cancers exhibit divergent prognostic architectures. Combined analyses may reduce risk stratification accuracy and obscure the true value of inflammatory biomarkers, supporting the development of tumor site–specific prognostic models in colorectal oncology.

## Introduction

Rectal cancer (RC) continues to be analyzed within the broader context of colorectal cancer (CRC) in a substantial number of clinical and translational studies, despite increasingly robust evidence indicating that it represents a biologically and clinically distinct malignant disease [1]. The differences between colon and rectal cancer are not limited to anatomical localization but encompass fundamental disparities in molecular carcinogenesis, patterns of genetic instability, expression of cell cycle regulators, and response to multimodal treatment [2–6]. Pronounced heterogeneity significantly compromises the applicability of prognostic models developed on pooled cohorts of CRC.

The biological specificity of RC is further underscored by unique therapeutic algorithms that include neoadjuvant chemoradiotherapy, total mesorectal excision, and, in selected patients, non-operative strategies such as the watch-and-wait approach [7–11]. These factors substantially modify disease course and survival outcomes, implying that prognostic markers must be validated specifically in populations of patients with RC.

Systemic inflammation represents a key element of tumor–host interaction. Contrary to the favorable prognostic significance of local immune infiltration, systemic inflammation reflects a more aggressive disease phenotype, reduced therapeutic efficacy and worse survival [12–16]. This response reflects complex biological processes, including cytokine activation, catabolic metabolism, and suppression of antitumor immunity [17, 18]. In recent years, combined hematological and biochemical indices integrating markers of inflammation and nutritional status such as the lymphocyte–albumin–neutrophil ratio (LANR), prognostic nutritional index (PNI), C-reactive protein–to–albumin ratio (CAR), and the Glasgow prognostic score (GPS) have demonstrated significant prognostic potential in gastrointestinal malignancies [13–16, 19–21]. However, the available evidence predominantly derives from studies analyzing heterogeneous CRC cohorts, often without stratification according to primary tumor location and mainly involving Asian populations [13, 14, 22, 23]. Consequently, it remains unclear to what extent these indices truly reflect the prognosis of RC as a distinct entity.

The aim of the present pilot study was to investigate the prognostic significance of systemic inflammatory and nutritional indices in a subgroup of patients with RC, with particular focus on their association with long-term oncological outcomes. The objective was not to compare colon and rectal cancer, but rather to evaluate whether prognostic inflammatory and nutritional markers identified in a previously studied CRC cohort retain their prognostic relevance when rectal adenocarcinoma is analyzed as a distinct disease entity. We hypothesized that these indices exhibit a specific and differential prognostic value in RC that cannot be adequately assessed within pooled CRC analyses.

## Materials and Methods

### Study design and study population

This study represents a secondary, hypothesis-driven analysis of a RC subgroup derived from a single-center cohort of patients surgically treated for colorectal adenocarcinoma at a tertiary care center between January and December 2017. The primary study design, inclusion and exclusion criteria, and data acquisition methodology have been described in detail in a previously published study by the same research group [24]. For the purpose of the present analysis, only adult patients with histopathologically confirmed rectal adenocarcinoma were included. Eligible patients had complete clinical, laboratory, and pathohistological data, as well as adequate postoperative follow-up. Patients with previously treated malignant diseases in a period shorter than 5 years before surgical intervention, as well as those with incomplete medical documentation or inadequate follow-up duration, were excluded from the analysis.

### Ethics approval and consent to participate

This study was conducted in accordance with the Declaration of Helsinki and approved by the Ethics Committee of the University Clinical Hospital Center “Zvezdara”. All patients provided written informed consent for participation in the study and for the use of medical records and biological samples for research purposes. Ethical approval procedures and data protection measures were fully consistent with those applied in the previously published study of the same cohort [24].

### Clinical and treatment data

Clinical data collected from medical records included patient sex, age, comorbidities, and length of hospital stay. Perioperative risk was assessed using the American Society of Anesthesiologists (ASA) score, while postoperative complications were classified according to the Clavien–Dindo classification. Information regarding neoadjuvant and adjuvant chemo- and/or radiotherapy was obtained from the Institute of Oncology and Radiology of Serbia.

### Laboratory assessment and inflammatory–nutritional indices

Preoperative peripheral blood samples were collected within 7 days prior to the planned surgical procedure. Standard hematological and biochemical analyses were performed in the central hospital laboratory using automated analyzers, in accordance with routine clinical practice. In addition to routine parameters, serum levels of C-reactive protein (CRP), albumin, and tumor markers carcinoembryonic antigen (CEA) and carbohydrate antigen 19-9 (CA 19-9) were measured. Based on the obtained laboratory values, combined hematological and biochemical indices and scores were calculated using standardized formulas previously described in the literature and validated in the prior study: neutrophil-to-lymphocyte ratio (NLR), monocyte-to-lymphocyte ratio (MLR), platelet-to-lymphocyte ratio (PLR), red cell distribution width-to-lymphocyte ratio (RLR), mean platelet volume-to-platelet ratio (MPR), CAR, PNI, modified Glasgow Prognostic Score (mGPS), and LANR [24].

### Histopathological features

All surgically removed preparations were processed in accordance with the valid standards of pathohistological analysis. Disease stage was determined using the TNM classification according to the criteria of the American Joint Committee on Cancer (AJCC) [25]. The analysis included determination of histological type and degree of tumor differentiation, presence of lymphovascular, venous and perineural invasion, assessment of resection margins, identification of tumor deposits, as well as assessment of tumor budding. The total number of isolated lymph nodes, the number of metastatic nodes, and their mutual relationship were recorded. Special attention was paid to parameters that reflect the host’s immune response in the tumor microenvironment, including lymphocyte infiltration within the tumor and peritumor lymphocyte reaction.

### Postoperative follow-up and outcome definitions

Postoperative follow-up was conducted in accordance with the National Guidelines of Good Clinical Practice for Colon and Rectal Cancer issued by the Ministry of Health of the Republic of Serbia [26]. Overall survival (OS) was defined as the time from surgical treatment to death from any cause. Disease-free survival (DFS) was defined as the interval from primary surgical treatment to clinically, biochemically, or radiologically confirmed disease recurrence.

Data on the death outcome were confirmed on the basis of hospital records and data from national death registries.

### Statistical analysis

Continuous variables are described by arithmetic mean and standard deviation or median with interquartile range, depending on the distribution; while categorical variables are presented as absolute numbers and percentages. The normality of the distribution was assessed using the Kolmogorov–Smirnov test. The Mann–Whitney test was used to compare continuous parameters between the studied groups, while the Chi-square test was used to analyze differences in the distribution of categorical variables. Survival was evaluated using the Kaplan–Meier method, with statistical evaluation of differences between curves using the log-rank test. The influence of individual clinical-pathological factors on OS and DFS was analyzed using Cox regression analysis in univariate and multivariate models. Variables demonstrating statistical significance (P < 0.05), as well as variables considered clinically relevant based on prior evidence that had borderline significance (P < 0.10) in univariate analyses, were considered for inclusion in multivariable models. Statistical significance was set at P < 0.05. Statistical analyses were conducted using IBM SPSS Statistics software, version 25.0. Survival graphs were plotted in Prism 9, GraphPad Software.

## Results

### Baseline clinicopathological characteristics of the overall CRC cohort and the RC subgroup

The present study builds upon our previously published cohort (Cuk et al, 2023) [24] by performing a predefined subgroup analysis focusing on RC as a distinct clinical entity, using the same analytical framework. A total of 111 patients with CRC, of which 29 patients had RC, were included in the analysis. The median postoperative follow-up time for both cohorts was 42 months (interquartile range, 24–48 months). Baseline demographic, clinical, laboratory, and pathohistological characteristics of patients with CRC and RC separately are presented in Table 1. The two groups were comparable with respect to sex distribution and age. Male patients predominated in both cohorts, accounting for 56.8% of patients in the CRC group and 62.1% in the RC group. Median age was similar between the CRC and RC cohorts (67 (interquartile range, 32–88) vs. 65 (interquartile range, 44–81) years). Regarding perioperative risk and overall health status, most patients in both cohorts had low to moderate ASA scores. In the CRC group, ASA 1 was recorded in 18 patients (16.2%), ASA 2 in 50 patients (45.0%), and ASA 3 in 43 patients (38.7%), with no patients classified as ASA 4 or ASA 5. A similar distribution was observed in the RC cohort, where ASA 2 was the most frequent category (14 patients; 48.3%), followed by ASA 1 (eight patients; 27.6%) and ASA 3 (seven patients; 24.1%). No patients in the RC group were assigned to ASA 4 or ASA 5 categories. In the CRC cohort, mGPS 0 was observed in 66 patients (59.5%), mGPS 1 in 31 patients (27.9%), and mGPS 2 in 14 patients (12.6%). In the RC cohort, the majority of patients were classified as mGPS 0 (22 patients; 75.9%). The remaining patients were categorized as having elevated mGPS values, with mGPS 1 and mGPS 2 analyzed as a combined category (seven patients; 24.1%), as shown in Table 1.

**Table 1:** Characteristics of Patients With Colorectal and Rectal Cancer

Characteristics of patients	Colorectal cancer, n (%)	Rectal cancer, n (%)
Sex		
Male	63 (56.8)	18 (62.1)
Age (years)^a^	67 (32−88)	65 (44–81)
ASA score		
1	18 (16.2)	8 (27.6)
2	50 (45.0)	14 (48.3)
3	43 (38.7)	7 (24.1)
4	0 (0)	0 (0)
5	0 (0)	0 (0)
Hematological/biochemical values and indexes^b^		
Leukocytes (× 10^9^/L)	7 (5.70−8.4)	6.00 (4.93–7.35)
Erythrocytes (× 10^9^/L)	4.51 (4.17−4.88)	4.54 (4.18–4.81)
Platelets (× 10^9^/L)	293 (243−377)	252 (193.5–374.5)
Neutrophils (× 10^9^/L)	4.72 (3.78−5.85)	3.95 (2.93–5.04)
Lymphocytes (× 10^9^/L)	1.47 (1.14−1.94)	1.44 (0.91–1.78)
Monocytes (× 10^9^/L)	0.35 (0.28−0.44)	0.32 (0.28–0.43)
Hemoglobin (g/dL)	12.10 (10.40−13.60)	12.40 (11.25–14.15)
Hematocrit (%)	37.70 (33.60−41.80)	38.50 (35.95–42.7)
RBC (RDW-CV) (%)	14.6 (13.3−17.4)	14.20 (13.20–16.35)
Serum albumin (g/L)	39 (35.00−42.00)	40 (36.00–43.00)
CRP (mg/mL)	4.80 (2.00−17.10)	3.50 (1.30–5.10)
CEA (ng/mL)	3.37 (2.02−9.11)	3.49 (2.21–6.40)
CA 19-9 (U/mL)	11.44 (6.28−28.46)	9.34 (6.15–31.40)
NLR	3.09 (2.21−4.54)	2.85 (1.77–4.62)
MLR	0.23 (0.18−0.32)	0.22 (0.17–0.36)
PLR	190.63 (141.60−276.14)	177.38 (140.94–267.73)
RLR	10.23 (7.62−14.1)	10.43 (7.83–15.07)
MPR	0.03 (0.02−0.04)	0.04 (0.03–0.05)
CAR	0.12 (0.05−0.45)	0.08 (0.03–0.13)
PNI	46.56 (42.30−50.85)	46.90 (42.33–51.30)
LANR	12.43 (7.78−17.76)	13.02 (8.86–20.88)
mGPS^c^		
0	66 (59.5)	22 (75.9)
1	31 (27.9)	7 (24.1)^d^
2	14 (12.6)	

^a^Data are shown as median (minimum–maximum). ^b^Data are shown as median (25−75 percentiles). ^c^mGPS 0 (CRP ≤ 10, Alb ≥ 35), mGPS 1 (CRP ≤ 10, Alb < 35 or CRP > 10, Alb ≥ 35), mGPS 2 (CRP > 10, Alb < 35). ^d^Patients were grouped as mGPS 1–2. ASA: American Society of Anesthesiology; RBC: red blood cell; RDW-CV: red cell distribution width–coefficient of variation; CRP: C-reactive protein; CEA: carcinoembryonic antigen; CA 19-9: carbohydrate antigen 19-9; NLR: neutrophil-to-lymphocyte ratio; MLR: monocyte-to-lymphocyte ratio; PLR: platelet-to-lymphocyte ratio; RLR: red cell distribution width-to-lymphocyte ratio; MPR: mean platelet volume-to-platelet ratio; PNI: prognostic nutritional index; CAR: C-reactive protein–to–albumin ratio; LANR: lymphocyte–albumin–neutrophil ratio; mGPS: modified Glasgow prognostic score; Alb: albumin.

### OS in the overall CRC cohort and the RC subgroup

Clinical and pathohistological characteristics in relation to OS in patients with CRC and RC are summarized in Table 2. In the CRC cohort, OS was significantly associated with a broad spectrum of systemic, hematological, biochemical, and tumor-related parameters. Patients who died during follow-up more frequently exhibited elevated markers of systemic inflammation and impaired nutritional status, including higher CRP levels, lower serum albumin concentrations, reduced hemoglobin and hematocrit values, increased red blood cell distribution width, and higher CEA levels. In addition, composite inflammatory and nutritional indices integrating these parameters such as CAR, PNI, LANR, PLR, and mGPS were significantly associated with survival outcomes.

**Table 2:** Clinical and Pathohistological Characteristics in Relation to Overall Survival in Patients With Colorectal and Rectal Cancer

	Colorectal cancer death			Rectal cancer death		
	No, n (%)	Yes, n (%)	P	No, n (%)	Yes, n (%)	P
Sex			0.300^a^			0.375^a^
Male	44 (60.3)	19 (50)		15 (68.2)	3 (42.9)	
Female	29 (39.7)	19 (50)		7 (31.8)	4 (57.1)	
ASA score			0.248^a^			0.935^a^
1	11 (15.1)	7 (18.4)		6 (27.3)	2 (28.6)	
2	37 (50.7)	13 (34.2)		11 (50)	3 (42.9)	
3	25 (34.2)	18 (47.4)		5 (22.7)	2 (28.6)	
4	—	—		—	—	
5	—	—		—	—	
Diabetes mellitus	21 (28.8)	8 (21.1)	0.380^a^	4 (18.2)	1 (14.3)	0.653^a^
Arterial hypertension	53 (72.6)	28 (73.7)	0.903^a^	14 (63.6)	5 (71.4)	0.541^a^
Leukocytes^c^	6.9 (5.80−8.10)	7.65 (5.6−8.8)	0.218^b^	5.93 (5.10–6.80)	6.9 (4.10–8.50)	0.610^b^
Erythrocytes^c^	4.55 (4.29−4.89)	4.3 (3.88−4.79)	0.059^b^	4.57 (4.36–4.81)	4.20 (3.93–4.58)	0.194^b^
Platelets^c^	282 (228−373)	317 (258−389)	0.090^b^	233 (192–321)	389 (314–428)	0.011*^b^
Neutrophils^c^	4.59 (3.78−5.66)	5.18 (3.84−7.12)	0.196^b^	3.89 (3.23–4.55)	4.58 (2.65–7.20)	0.610^b^
Lymphocytes^c^	1.54 (1.25−1.94)	1.33 (1.05−1.84)	0.254^b^	1.49 (1.03–1.78)	0.99 (0.88–1.78)	0.475^b^
Monocytes^c^	0.34 (0.29−0.42)	0.35 (0.26−0.48)	0.751^b^	0.32 (0.28–0.38)	0.34 (0.22–0.44)	0.878^b^
NLR^c^	2.89 (2.22−4.44)	3.73 (2.21−4.8)	0.160^b^	2.84 (1.72–4.44)	3.01 (2.0–8.57)	0.508^b^
MLR^c^	0.22 (0.17−0.29)	0.25 (0.19−0.35)	0.234^b^	0.21 (0.16–0.37)	0.27 (0.21–0.35)	0.359^b^
PLR^c^	188.3 (138.14−235.62)	211.64 (153.26−324.6)	0.056*^b^	154.9 (131.95–109.5)	259.2 (183.84–465.2)	0.013^b^
CRP^c^	3.5 (1.7−9.8)	10.3 (4−34.9)	0.001*^b^	3.0 (1.3–4.0)	6.7 (1.3–34.9)	0.132^b^
Serum albumin^c^	40 (36−42)	37 (33−41)	0.005*^b^	41 (37–43)	36 (35–42)	0.209^b^
Hemoglobin^c^	12.4 (10.8−13.7)	11 (9.4−12.9)	0.037*^b^	13.1 (11.8–14.2)	11.0 (8.6–12.8)	0.059^b^
Hematocrit^c^	38.6 (35.2−41.9)	35.2 (31.9−41.7)	0.027*^b^	39.7 (37.5–43.0)	35.8 (29.2–38.1)	0.041*^b^
RBC (RDW-CV)^ c^	14.2 (13.2−16.7	14.9 (14−19.8)	0.041*^b^	13.5 (13.1–15.8)	14.9 (14.6–19.4)	0.039*^b^
CAR^c^	0.08 (0.04−0.24)	0.257 (0.11−0.85)	0.001*^b^	0.077 (0.033–0.093)	0.164 (0.037–0.851)	0.093^b^
PNI^c^	47.3 (43.8−51.25)	54.1 (39.6−48.6)	0.013*^b^	46.63 (43.5–51.45)	45.2 (39.4–48.6)	0.359^b^
LANR^c^	13.27 (8.86−18.9)	9.85 (7.09−16.28)	0.043*^b^	13.58 (8.86–22.52)	11.62 (4.78–17.92)	0.241^b^
CEA^c^	2.99 (1.88−6.48)	5.2 (2.41−10.05)	0.030*^b^	3.5 (2.30–9.86)	3.23 (2.10–6.11)	0.460^b^
CA 19−9^c^	10.41 (6.4−27.59)	14.33 (6.22−37.16)	0.230^b^	9.21 (6.52–31.10)	10.95 (4.98–37.16)	0.541^b^
mGPS			0.003*^a^			
0	50 (68.5)	16 (42.1)		17 (77.3)^d^	5 (71.4)^d^	0.753^a^
1	19 (26)	12 (31.6)		5 (22.7)^d^	2 (28.6)^d^	
2	4 (5.5)	10 (26.3)				
Max diameter of tumor^c^	40 (30−55)	40 (30−55)	0.741^a^	40 (25–55)	45 (35–65)	0.271^a^
T status			0.032*			0.028*
T1/T2	28 (38.4)	7 (18.4)		10 (45.5)	0 (0)	
T3/T4	45 (61.6)	31 (81.6)		12 (54.5)	7 (100)	
TNM stage			0.009*^a^			0.374^a^
I/II	46 (63)	14 (36.8)		13 (59.1)	3 (42.9)	
III/IV	27 (37)	24 (63.2)		9 (40.9)	4 (57.1)	
Number of lymph nodes^c^	17 (12−21)	14 (12−17)	0.120^a^	13 (11–18)	11 (10–18)	0.329^a^
Positive lymph nodes^c^	0 (0−1)	2 (0−5)	0.003*^b^	0 (0–1)	0 (0–6)	0.489^b^
Lymph node ratio (LNR)	0 (0−0.048)	0.113 (0−0.353)	0.001*^b^	0 (0−0.148)	0.113 (0−0.268)	0.417^b^
Tumor configuration			0.262^a^			0.353^a^
Exophytic	29 (39.7)	11 (28.9)		7 (31.8)	1 (14.3)	
Endophytic	44 (60.3)	27 (71.1)		15 (68.2)	6 (85.7)	
TIL			0.380^a^			0.545^a^
Without/easy to moderate	52 (71.2)	30 (78.9)		17 (77.3)	6 (85.7)	
Expressed	21 (28.8)	8 (21.1)		5 (2.7)	1 (14.3)	
PTL response			0.019*^a^			0.647^a^
Without	14 (19.2)	12 (31.6)		6 (27.3)^e^	2 (28.6)^e^	
Easy to moderate	43 (58.9)	25 (65.8)		16 (72.7)^e^	5 (71.4)^e^	
Expressed	16 (21.9)	1 (2.6)				
Mucosal component of the tumor			0.401^a^			0.459^a^
Yes	23 (31.5)	15 (39.5)		7 (31.8)	3 (42.9)	
No	50 (68.5)	23 (60.5)		15 (68.2)	4 (57.1)	
Lymphovascular invasion			0.205^a^			0.758^a^
Yes	45 (61.6)	28 (73.7)		14 (63.6)	4 (57.1)	
No	28 (38.4)	10 (26.3)		8 (36.4)	3 (42.9)	
Venous invasion			0.015*^a^			0.009*^a^
Yes	0 (0)	3 (7.9)		0 (0.0)	2 (28.6)	
No	73 (100)	35 (92.1)		22 (100)	5 (71.4)	
Perineural invasion			0.001*^a^			0.003^a^
Yes	10 (13.7)	16 (42.1)		2 (9.1)	5 (71.4)	
No	63 (86.3)	22 (57.9)		20 (90.9)	2 (28.6)	
Tumor deposits			0.001*^a^			0.184^a^
Yes	7 (9.6)	13 (34.2)		4 (18.2)	3 (42.9)	
No	66 (90.4)	25 (65.8)		18 (81.8)	4 (57.1)	
Tumor budding			0.037*^a^			0.121^a^
Yes	55 (75.3)	34 (91.9)		16 (72.7)	7 (100.0)	
No	18 (24.7)	3 (8.1)		6 (27.3)	0 (0)	
Tumor growth			0.113^a^			0.484^a^
Expansive	32 (44.4)	11 (28.9)		6 (27.3)	1 (14.3)	
Infiltrative	40 (55.6)	27 (71.1)		16 (72.7)	6 (85.7)	
C–D classification			0.003*^a^			0.159^a^
I	38 (52.1)	8 (21.1)		13 (59.1)^f^	2 (28.6)^f^	
II	30 (41.1)	22 (57.9)		9 (40.9)^f^	5 (71.4)^f^	
III, IV, V	5 (6.8)	8 (21.1)				
Adjuvant CT			0.644^a^			0.631^a^
Yes	20 (27.4)	12 (31.6)		9 (40.9)	3 (42.9)	
No	53 (72.6)	26 (68.4)		13 (59.1)	4 (57.1)	
Neoadjuvant CRT			0.619^a^			0.455^a^
Yes	4 (5.5)	3 (7.9)		4 (18.2)	2 (28.6)	
No	69 (94.5)	35 (92.1)		18 (81.8)	5 (71.4)	

^*^P values indicate statistical significance (P < 0.05). ^a^P values were calculated by the Chi-square test. ^b^P values were calculated by the Mann–Whitney test. ^c^Data are shown as median (25−75 percentiles). ^d^Patients were categorized into two groups as mGPS 0 and mGPS 1–2. ^e^Patients were categorized into two groups as without and easy to moderate/expressed PTL response. ^f^Patients were categorized into two groups as C–D classification I and C–D II, III, IV, V. TIL: tumor-infiltrating lymphocytes; PTL: peritumoral lymphocytic; C–D classification: Clavien–Dindo classification; CT: chemotherapy; CRT: chemoradiotherapy; ASA: American Society of Anesthesiology; RBC: red blood cell; RDW-CV: red cell distribution width–coefficient of variation; CRP: C-reactive protein; CEA: carcinoembryonic antigen; CA 19-9: carbohydrate antigen 19-9; NLR: neutrophil-to-lymphocyte ratio; MLR: monocyte-to-lymphocyte ratio; PLR: platelet-to-lymphocyte ratio; LANR: lymphocyte–albumin–neutrophil ratio; PNI: prognostic nutritional index; CAR: C-reactive protein–to–albumin ratio; mGPS: modified Glasgow prognostic score.

With regard to tumor-related pathological features in the CRC cohort, fatal outcomes were more frequently observed in patients with advanced T status (T3/T4), higher TNM stage (III/IV), increased lymph node involvement, higher lymph node ratio (LNR), and adverse histopathological characteristics, including perineural invasion, venous invasion, tumor deposits, tumor budding, weaker peritumoral lymphocytic response, and higher grades of postoperative complications according to the Clavien–Dindo classification.

In contrast, when analyzed separately in the RC subgroup, inflammatory and nutritional parameters, as well as their composite indices, showed limited associations with OS. Tumor-related pathological characteristics demonstrated stronger associations with outcome in this subgroup, particularly advanced T status and the presence of perineural invasion. Other pathological variables, including TNM stage, lymph node involvement, lymphovascular invasion, tumor deposits, tumor budding, tumor growth pattern, and postoperative complication grade, did not show statistically significant associations with survival.

Parameters that demonstrated statistical significance in relation to OS in both CRC and RC cohorts in Table 2 were subsequently subjected to Cox regression analysis, with the results presented in Table 3. In the CRC cohort, multiple variables showed associations with OS in univariate analysis. In line with previously published analyses from this cohort (Cuk et al, 2023) [24], multivariate Cox regression analysis identified LNR, presence of tumor deposits, higher grades of postoperative complications according to the Clavien–Dindo classification (grades III–V), and higher mGPS as significant predictors of poorer OS.

**Table 3:** Cox Regression Analysis for Overall Survival in Colorectal and Rectal Cancer Patients

Variable	Univariate analysis			Multivariate analysis		
	HR	95% CI	P	HR	95% CI	P
Colorectal cancer						
TNM stage (III/IV)^a^	2.430	1.254–4.711	0.009*		—	—
LNR	16.706	4.890–57.074	< 0.001*	6.862	1.635–28.808	0.009*
PTL response	0.531	0.317–0.890	0.016*	—	—	—
Perineural invasion (presence)	2.988	1.563–5.709	0.001*	—	—	—
Tumor deposits (presence)	3.254	1.652–6.409	0.001*	3.089	1.447–6.593	0.004*
Tumor budding (presence)	3.233	0.992–10.540	0.052*	—	—	—
C–D classification (gradus III, IV, V)	2.528	1.574–4.061	< 0.001*	2.609	1.437–4.737	0.002*
Hemoglobin (g/dL)	0.844	0.723–0.985	0.031*	—	—	—
Hematocrit (%)	0.938	0.885–0.994	0.030*	—	—	—
CRP (mg/L)	1.009	1.003–1.016	0.006*	—	—	—
Serum albumin (g/L)	0.897	0.843–0.955	0.001*	—	—	—
CEA (ng/mL)	1.010	1.000–1.019	0.041*	—	—	—
PLR	1.002	1.00–1.005	0.045*	—	—	—
LANR	0.946	0.898–0.996	0.035*	—	—	—
CAR	1.335	1.102–1.617	0.003*	—	—	—
PNI	0.924	0.877–0.974	0.003*		—	—
mGPS 2	2.145	1.431–3.215	< 0.001*	2.188	1.413–3.387	< 0.001*
Rectal cancer						
Perineural invasion (presence)	12.389	2.345–65.452	0.003*	25.125	2.928–218.620	0.003*
PLR	1.003	1.000–1.007	0.060	1.007	1.001–1.013	0.025*

^*^P values indicate statistical significance (P < 0.05). ^a^TNM stage adopted binary classification (I/II vs. III/IV). HR: hazard ratio; CI: confidence interval; LNR: lymph node ratio; PTL: peritumoral lymphocytic; C–D classification: Clavien–Dindo classification; CRP: C-reactive protein; CEA: carcinoembryonic antigen; PLR: platelet-to-lymphocyte ratio; LANR: lymphocyte–albumin–neutrophil ratio; PNI: prognostic nutritional index; CAR: C-reactive protein–to–albumin ratio; mGPS: modified Glasgow prognostic score.

However, when analyzed separately in the RC subgroup, fewer variables demonstrated prognostic relevance. Variables with a borderline significance in univariate analysis were included in the multivariate analysis, if they were clinically relevant; such were the presence of tumor budding (with P = 0.052 in CRC) and platelets to lymphocyte ratio (with P = 0.06 in RC). While several clinicopathological parameters were associated with OS in univariate analysis, multivariate Cox regression analysis identified perineural invasion and PLR as significant predictors of OS in the RC subgroup. The presence of perineural invasion was associated with a significantly increased risk of mortality (hazard ratio (HR) = 25.125, P = 0.003), while higher PLR values were also significantly associated with worse survival outcomes (HR = 1.007, P = 0.025).

**Figure 1:**
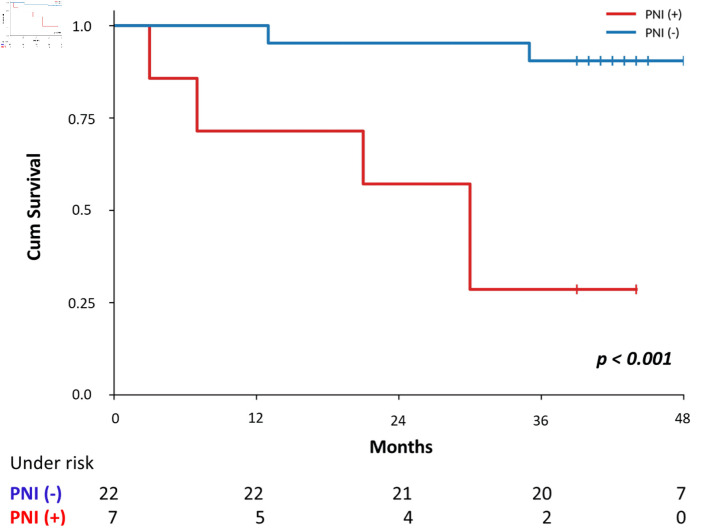
Kaplan–Meier curves for overall survival in rectal cancer patients grouped by perineural invasion. PNI: prognostic nutritional index.

Kaplan–Meier curves for OS in relation to the degree of postoperative complications according to the Clavien–Dindo classification, the mGPS and the presence of tumor deposits in a cohort of CRC patients were previously published (Cuk et al, 2023) [24]. In this study, a survival analysis is presented for patients with RC, allowing a direct comparison between the two cohorts. Kaplan–Meier survival analysis for RC patients demonstrated a significant difference in OS according to the presence of perineural invasion (Fig. 1). A total of 29 patients were included in the analysis; during the follow-up period, 22 patients survived, while seven deaths were recorded. Among the surviving patients, perineural invasion was present in two patients (9.1%), whereas among patients who died during follow-up, perineural invasion was observed in five patients (71.4%). Patients with perineural invasion exhibited shorter OS compared with those without perineural invasion (25.6 ± 5.7 months vs. 45.8 ± 1.6 months). The median OS in patients with perineural invasion was 30 months (interquartile range, 19.5–40.5 months). The log-rank test demonstrated a statistically significant difference in OS between the two groups (log-rank = 14.212; P < 0.001). While these results are from a small single center cohort and should be considered exploratory, they lay a ground for examination in larger independent cohorts.

### DFS in the CRC cohort and the RC subgroup

Clinicopathological, hematological, biochemical, and treatment-related characteristics in relation to DFS in patients with CRC and RC are summarized in Table 4.

**Table 4:** Clinical and Pathohistological Characteristics in Relation to Disease-Free Survival in Patients With Colorectal and Rectal Cancer

	Colorectal cancer relapse			Rectal cancer relapse		
	No, n (%)	Yes, n (%)	P	No, n (%)	Yes, n (%)	P
Sex			0.892			0.758
Male	41 (57.7)	16 (59.3)		14 (63.6)	4 (57.1)	
Female	30 (42.3)	11 (40.7)		8 (36.4)	3 (42.9)	
ASA score			0.032*^a^			0.196
1	9 (12.7)	8 (29.6)		6 (27.3)	2 (28.6)	
2	31 (43.7)	14 (51.9)		9 (40.9)	5 (71.4)	
3	31 (43.7)	5 (18.5)		7 (31.8)	0 (0.0)	
4	—	—		—	—	
5	—	—		—	—	
Diabetes mellitus	21 (29.6)	5 (18.5)	0.268^a^	5 (22.7)	0 (0.0)	0.166
Arterial hypertension	53 (74.6)	17 (63.0)	0.253^a^	14 (63.6)	5 (71.4)	0.706
Leukocytes^c^	7 (5.88–8.1)	7.7 (5.4–8.7)	0.811	6.05 (5.10–6.90)	5.40 (4.20–8.50)	0.980
Erythrocytes^c^	4.48 (4.17–4.86)	4.57 (4.14–5.14)	0.375	4.56 (4.19–4.81)	4.36 (3.93–4.80)	0.400
Platelets^c^	282 (228–375)	285 (230–392)	0.431	249 (198–350)	374 (177–428)	0.541
Neutrophils^c^	4.68 (3.86–5.77)	5.05 (3.08–6.58)	0.880	4.03 (3.23–4.58)	3.57 (2.19–7.20)	0.878
Lymphocytes^c^	1.51 (1.25–1.94)	1.56 (1–2)	0.990	1.47 (0.90–1.78)	1.25 (0.92–1.78)	0.899
Monocytes^c^	0.36 (0.29–0.44)	0.32 (0.25–0.39)	0.183	0.33 (0.29–0.44)	0.29 (0.19–0.41)	0.146
NLR^c^	2.9 (2.25–4.52)	2.9 (1.83–4.51)	0.833^a^	2.93 (1.82–4.44)	2.34 (1.70–8.57)	0.799^a^
MLR^c^	0.22 (0.18–0.32)	0.22 (0.17–0.27)	0.278^a^	0.22 (0.16–0.39)	0.22 (0.19–0.27)	0.646^a^
PLR^c^	187.5 (140.3–245.03)	193.88 (138.97–322.45)	0.559^a^	164.7 (140.3–259.3)	183.8 (141.6–465.2)	0.575^a^
CRP^c^	0.03 (0.03–0.43)	0.03 (0.02–0.043)	0.933^a^	3.3 (1.3–4.1)	3.5 (1.3–34.9)	0.371
Serum albumin^c^	40 (36–42)	41 (35–42)	1.000	40 (37–43)	41 (35–42)	0.626
Hemoglobin^c^	12 (10–13.6)	12.3 (10.4–13.7)	0.535	12.9 (11.7–14.2)	12.3 (10.4–14.1)	0.320
Hematocrit^c^	37.7 (33.6–41.7)	38.7 (32.6–42.9)	0.580	38.6 (37.4–43.0)	36.1 (32.4–42.3)	0.386
RBC (RDW-CV)^c^	14.7 (13.3–17.4)	14.4 (13.2–16.7)	0.404	14.1 (13.1–16.3)	14.6 (13.4–16.7)	0.429
CAR^c^	0.09 (0.05–0.32)	0.16 (0.04–0.38)	0.611^a^	0.079 (0.036–0.11)	0.097 (0.031–0.85)	0.333^a^
PNI^c^	46.7 (43.3–51.15)	47 (43.1–50.85)	0.849^a^	45.53 (42.5–51.45)	45.9 (41.25–50.85)	0.919^a^
LANR^c^	13.27 (8.69–16.89)	12.43 (7.72–19)	0.852^a^	13.0 (8.86–22.43)	17.62 (4.78–18.0)	0.919^a^
CEA^c^	2.76 (1.88–6.31)	3.59 (1.94–12.74)	0.361	3.37 (2.30–6.31)	6.11 (2.10–20.05)	0.491
CA 19−9^c^	10.92 (6.28–20.84)	12.01 (6.22–41.8)	0.324	9.21 (6.52–31.10)	14.07 (4.98–37.16)	0.507
mGPS			0.700^b^			0.184^b^
0	46 (64.8)	15 (55.6)		18 (81.8)^d^	4 (57.1)^d^	
1	19 (26.8)	9 (33.3)		4 (18.2)^d^	3 (42.9)^d^	
2	6 (8.5)	3 (11.1)				
TNM stage						0.452
I/II	50 (70.4)	8 (29.6)	< 0.001*	13 (59.1)	3 (42.9)	
III/IV	21 (29.6)	19 (70.4)		9 (40.9)	4 (57.1)	
Tumor grade			0.193		0.631	
G1	10 (14.1)	5 (18.5)		1 (4.5)	2 (28.6)	
G2	59 (83.1)	19 (70.4)		20 (90.9)	5 (71.4)	
G3	2 (2.8)	3 (11.1)		1 (4.5)	0 (0.0)	
Lymph nodes^c^			0.009*			0.374
N0	49 (69)	10 (37.0)		13 (59.1)	3 (42.9)	
N1	16 (22.5)	10 (37.0)		9 (40.9)	4 (57.1)	
N2	6 (8.5)	7 (25.8)				
Tumor configuration			0.731			0.366
Exophytic	29 (40.8)	10 (37.0)		7 (31.8)	1 (14.3)	
Endophytic	42 (59.2)	17 (63.0)		15 (68.2)	6 (85.7)	
TIL			0.520			0.631
Without/easy to moderate	52 (73.2)	18 (66.7)		17 (77.3)	6 (85.7)	
Expressed	19 (26.8)	9 (33.3)		5 (22.7)	1 (14.3)	
PTL response			0.029*			0.974
Without	11 (15.5)	9 (33.3)		6 (27.3)^e^	2 (28.6)^e^	
Easy to moderate	44 (62.0)	17 (63.0)		16 (72.7)^e^	5 (71.4)^e^	
Expressed	16 (22.5)	1 (3.7)				
Mucosal component of the tumor			0.292^a^			0.706
Yes	21 (29.6)	11 (40.7)		8 (36.4)	2 (28.6)	
No	50 (70.4)	16 (59.3)		14 (63.6)	5 (71.4)	
Lymphovascular invasion			0.038*^a^			0.758
Yes	39 (54.9)	21 (77.8)		14 (63.6)	4 (57.1)	
No	32 (45.1)	6 (22.2)		8 (36.4)	3 (42.9)	
Venous invasion			0.020*^a^			
Yes	0 (0)	2 (7.4)		1 (4.5)	1 (14.3)	0.376
No	71 (100.0)	25 (92.6)		21 (95.5)	6 (85.7)	
Perineural invasion			0.018*^a^			0.184
Yes	9 (12.7)	9 (33.3)		4 (18.2)	3 (42.9)	
No	62 (87.3)	18 (66.7)		18 (81.8)	4 (57.1)	
Tumor deposits			0.001*^a^			0.753
Yes	5 (7.0)	9 (33.3)		5 (22.7)	2 (28.6)	
No	66 (93)	18 (66.7)		17 (77.3)	5 (71.4)	
Tumor budding			0.838^a^			0.121
Yes	56 (78.9)	21 (80.8)		16 (72.7)	7 (100)	
No	15 (21.1)	5 (19.2)		6 (27.3)	0 (0)	
Tumor growth			0.752			0.484
Expansive	31 (44.3)	11 (40.7)		6 (27.3)	1 (14.3)	
Infiltrative	39 (55.7)	16 (59.3)		16 (72.7)	6 (85.7)	
C–D classification			0.598			0.590
I	32 (45.1)	11 (40.7)		12 (54.5)^f^	3 (42.9)^f^	
II	33 (46.5)	15 (55.6)		10 (45.5)^f^	4 (57.1)^f^	
III, IV, V	6 (8.5)	1 (3.7)				
Adjuvant CT			< 0.001*			0.331
Yes	13 (18.3)	15 (55.6)		8 (36.4)	4 (57.1)	
No	58 (81.7)	12 (44.4)		14 (63.6)	3 (42.9)	
Neoadjuvant CRT			0.744			0.631
Yes	4 (5.6)	2 (7.4)		5 (22.7)	1 (14.3)	
No	67 (94.4)	25 (96.2)		17 (77.3)	6 (85.7)	

^a^P values were calculated by the Chi-square test. ^b^P values were calculated by the Mann–Whitney test. ^c^Data are shown as median (25−75 percentiles). ^d^Patients were categorized into two groups as mGPS 0 and mGPS 1-2. ^e^Patients were categorized into two groups as without and easy to moderate/expressed PTL response. ^f^Patients were categorized into two groups as C–D classification I and C–D II, III, IV, V. ^*^P values indicate statistical significance (P < 0.05). TIL: tumor-infiltrating lymphocytes; PTL: peritumoral lymphocytic; C–D classification: Clavien–Dindo classification; CT: chemotherapy; CRT: chemoradiotherapy; ASA: American Society of Anesthesiology; RBC: red blood cell; RDW-CV: red cell distribution width–coefficient of variation; CRP: C-reactive protein; CEA: carcinoembryonic antigen; CA 19-9: carbohydrate antigen 19-9; NLR: neutrophil-to-lymphocyte ratio; MLR: monocyte-to-lymphocyte ratio; PLR: platelet-to-lymphocyte ratio; LANR: lymphocyte–albumin–neutrophil ratio; PNI: prognostic nutritional index; CAR: C-reactive protein–to–albumin ratio; mGPS: modified Glasgow prognostic score.

In the CRC cohort, disease recurrence was significantly associated with several clinical and pathological variables. Higher ASA scores were more frequently observed among patients who developed disease relapse (P = 0.032). Advanced tumor stage was strongly associated with recurrence, with patients presenting with TNM stage III/IV experiencing relapses more frequently compared with those in stage I/II (P < 0.001). Lymph node involvement was also significantly related to recurrence, with higher rates of relapse observed in patients with nodal metastases (P = 0.009).

Regarding tumor-related pathological features, disease recurrence in the CRC cohort was more common in patients with lymphovascular invasion (P = 0.038), venous invasion (P = 0.020), perineural invasion (P = 0.018), and the presence of tumor deposits (P = 0.001). In addition, a weaker peritumoral lymphocytic response was associated with higher relapse rates (P = 0.029). Patients receiving adjuvant chemotherapy also differed significantly with respect to recurrence status (P < 0.001). In contrast, systemic inflammatory markers, nutritional parameters, and composite inflammatory indices did not demonstrate significant associations with DFS in this cohort.

None of these associations retained statistical significance when RC patients were analyzed separately. In the RC cohort, no significant differences in relapse rates were observed in relation to demographic characteristics, ASA score, systemic inflammatory or nutritional parameters, or composite indices. Tumor-related pathological characteristics, including TNM stage, lymph node status, tumor invasion patterns, and adverse histopathological features, did not demonstrate statistically significant associations with disease recurrence in this cohort. Similarly, neoadjuvant and adjuvant treatment modalities were not significantly associated with DFS.

Results of univariate and multivariate Cox regression analyses for DFS in the CRC cohort have been reported previously (Cuk et al, 2023) [24].

For completeness and comparative purposes, the corresponding analyses are summarized in Table 5. In the CRC cohort, univariate analysis demonstrated that advanced TNM stage (III/IV), weaker peritumoral lymphocytic (PTL) response, presence of perineural invasion, and presence of tumor deposits were significantly associated with an increased risk of disease recurrence. In multivariate analysis, TNM stage III/IV (HR = 1.888, P = 0.042), weaker PTL response (HR = 0.391, P = 0.005), and presence of tumor deposits (HR = 3.049, P = 0.018) remained significantly associated with DFS.

**Table 5:** Clinical and Pathohistological Characteristics in Relation to Disease-Free Survival in Patients With Colorectal and Rectal Cancer

Variable	Univariate analysis			Multivariate analysis		
	HR	95% CI	P	HR	95% CI	P
Colorectal cancer						
TNM stage (III/IV)	2.486	1.390–4.445	0.002*	1.888	1.024–3.481	0.042*
PTL response (presence)	0.465	0.252–0.858	0.014*	0.391	0.196–0.780	0.005*
Perineural invasion (presence)	2.374	1.064–5.299	0.035*	—	—	—
Tumor deposits (presence)	4.194	1.869–9.411	0.001*	3.049	1.206–7.706	0.018*
Rectal cancer	—			—		

*P values indicate statistical significance (P < 0.05). HR: hazard ratio; CI: confidence interval; PTL: peritumoral lymphocytic.

Kaplan–Meier DFS curve in patients relative to the TNM stage, presence of tumor deposits and peritumoral lymphocytic response in the CRC cohort has been reported previously (Cuk et al, 2023) [24].

## Discussion

The findings of this study clearly demonstrate that CRC, when analyzed as a single entity, and RC, when considered separately, exhibit fundamentally divergent prognostic patterns. Although colon and rectal cancer are traditionally grouped together into CRC in clinical and epidemiological research, our results suggest that such an approach may obscure important biological differences and lead to inaccurate prognostic interpretation, particularly when prognostic models rely on peripheral hematologic biomarkers.

While the results related to the cohort of patients with CRC were previously published (Cuk et al, 2023) [24], in this paper, they are presented for comparative analysis with adenocarcinoma of the rectum. Rather than considering each individual marker separately, this analysis emphasizes the overall prognostic structure and dominant patterns that distinguish CRC as a group and RC as a separate entity. Overall, Cox regression analysis showed that the prognostic patterns differed significantly between the two groups. In the colorectal cohort, a more severe degree of postoperative complications of the Clavien–Dindo classification system (grades III, IV, V), a higher lymphnodal ratio, the presence of tumor deposits, and mGPS2 were good indicators of OS, while TNM stage, a weaker peritumoral lymphocyte response, and the presence of tumor deposits were significant indicators of the survival period without disease relapse. In contrast, in rectal adenocarcinoma subgroup, only perineural invasion emerged as a dominant and strong prognostic factor for OS. The PLR also showed statistical significance in relation to OS, but with a much smaller prognostic contribution, while the influence of other systemic inflammatory and pathohistological parameters was limited. These findings suggest that biological mechanisms of progression and determinants of outcome differ between CRC and RC.

In the colorectal cohort, marked systemic inflammation and impaired nutritional status, defined by elevated CRP and decreased albumin (mGPS 2), as well as anemia and elevated CEA, were consistently associated with worse outcomes. Similarly, composite inflammatory/nutritional indices (PLR, LANR, CAR and PNI) retained strong prognostic value. This pattern clearly indicates that systemic inflammation and nutritional status represent key mechanisms influencing CRC progression. Elevated CRP and decreased albumin reflect enhanced cytokine activity and a catabolic state, whereas indices based on platelet, neutrophil, and lymphocyte ratios capture alterations in the host immune response [13–16]. These mechanisms, well documented in previous studies, probably explain why inflammatory indices such as NLR, LANR and CAR remain reliable prognostic indicators in CRC [27–29]. Contrary to findings in the colorectal cohort, inflammatory and nutritional indices did not show a comparable prognostic value in patients with RC. Even parameters that were strong predictors of outcome in the CRC group did not retain statistical significance in the rectal subgroup (Table 3). This finding is particularly significant considering that certain studies, especially in patients treated with preoperative chemoradiotherapy [30], indicate the potential prognostic value of inflammatory markers in RC. It is possible that the inflammatory response in RC is largely determined by the therapeutic context. In our cohort, in which the majority of patients underwent primary surgical treatment without neoadjuvant therapy, rectal tumors appeared to induce a significantly weaker systemic inflammatory response compared with colorectal tumors considered as a single entity. Given that the secondary subgroup analysis has a small number of patients, these results need further confirmation in larger independent cohorts. However, our findings that prognostic value of inflammatory markers differs in RC compared with CRC as a whole could have important clinical implications.

The PLR, in relation to OS, in the pooled CRC cohort showed limited prognostic value, with significance present only in univariate analysis (HR = 1.002; 95% confidence interval (CI), 1.00–1.005; P = 0.045) (Table 3) and no independent effect after adjustment for other factors. In contrast, among patients with RC, this parameter remained independently associated with OS (HR = 1.007; 95% CI, 1.001–1.013; P = 0.025) (Table 3). These findings should be interpreted in the context of previously published data. The results of some studies indicated an association of elevated PLR with CRC progression [31], while earlier reports indicated its independent prognostic value for recurrence-free survival in patients with metastatic colorectal adenocarcinoma receiving adjuvant therapy [32]. On the contrary, some studies did not confirm its prognostic significance but showed the association of the value of this index with specific molecular changes, such as *PIK3CA* and *TP53* mutations [33]. A large meta-analysis that included 14,205 patients identified a high preoperative PLR as a significant predictor of survival in operated patients with rectal adenocarcinoma [34]. Lee et al determined that both the initial values of PLR, as well as its changes during and after chemoradiotherapy, were significantly related to the degree of pathohistological advancement of the disease, the effectiveness of the therapeutic response and the probability of recurrence in patients with locally advanced RC [35]. However, recent research has questioned its independent prognostic influence in RC [36]. In the context of heterogeneous findings from the literature, our results are partially consistent with previous data: PLR was shown to be a significant predictor of OS in RC, but not in the pooled CRC cohort, nor was it associated with recurrence-free survival. These findings further indicate the need to consider RC separately when evaluating systemic inflammatory biomarkers.

Perineural invasion, defined as tumor infiltration into the nerve sheath or perineural space involving at least one-third of the nerve’s circumference [37], was associated with an approximately threefold increased risk of death and a twofold increased risk of disease recurrence in our pooled cohort of CRC patients. This finding confirms its importance as a marker of more aggressive tumor behavior but in the context of CRC it acts as one of several unfavorable prognostic parameters within the complex interaction of other pathohistological parameters, systemic inflammation and nutritional status. However, in the subgroup of patients with adenocarcinoma of the rectum, perineural invasion stood out as the dominant and only significant pathohistological predictor of OS, with a markedly elevated HR. It should be emphasized that the HR recorded for perineural invasion in the cohort of patients with RC was accompanied by a wide CI. This most likely reflects the limited sample size and relatively small number of events in this subgroup, which may affect the stability of the multivariate model. Although the strength of the observed association suggests a clinically significant effect, these findings should be interpreted with caution and confirmed in larger, independent cohorts. The prognostic significance of perineural invasion was also confirmed in the 2016 meta-analysis by Knijn et al, which showed that the presence of perineural invasion significantly increases the risk of death in patients with colorectal adenocarcinoma, including RC (HR = 1.7; 95% CI, 1.48–1.86) [38]. Perineural invasion is therefore consistently recognized as a marker of aggressive tumor biological behavior, especially in rectal adenocarcinoma, where it is associated with a worse prognosis and a higher risk of recurrence [39]. Our results are consistent with these observations with regard to OS but differ with respect to the impact of perineural invasion on disease relapse in the rectal cohort. The frequency of perineural invasion in our cohort (24.1%) was comparable to previously published data [40–42]. Additional data from the literature indicate that perineural and lymphovascular invasion significantly modify the oncological outcome in patients with RC treated with neoadjuvant chemoradiotherapy [40]. Also, perineural invasion is associated with a lower 5-year relapse-free survival rate and remains an unfavorable prognostic factor even in patients with negative lymph nodes (ypN0) [43]. The differences between our study and some previous studies probably reflect the heterogeneity of populations, therapeutic approaches, and methodological evaluation criteria. Nevertheless, the consistent direction and magnitude of the effect further support the central role of local invasive patterns in determining the prognosis of RC. In contrast to the colorectal cohort where the prognosis was the result of the combined action of systemic and tumor factors, RC outcomes seem to be predominantly driven by patterns of local invasion.

This study has several limitations. First, its single-center design may limit the generalizability of the findings. Second, the relatively small sample size of the RC subgroup and the limited number of events may have influenced the stability of multivariate estimates, particularly for perineural invasion. Although the Kaplan–Meier analysis was supplemented with a number-at-risk table, the limited number of patients remaining at risk toward the end of the follow-up period may have affected the precision of the survival estimates. Future multicenter studies with larger cohorts are warranted to confirm these observations.

### Conclusions

The results of this study indicate that CRC should not be analyzed as a single prognostic entity, especially when evaluating systemic inflammatory and nutritional biomarkers. The observed divergent prognostic structure between rectal tumor site when analyzed alone and the CRC analyzed as a single entity highlights the need for site-specific risk stratification models. Such methodological precision can improve the accuracy of prognosis assessment and contribute to a more rational development of personalized therapeutic strategies in colorectal oncology. Our observations lay a ground for larger prospective studies that would lead to improved predictor precision.

## Data Availability

The data supporting the findings of this study are available from the corresponding author upon reasonable request.
